# Genome-Wide Compensatory Changes Accompany Drug- Selected Mutations in the *Plasmodium falciparum crt* Gene

**DOI:** 10.1371/journal.pone.0002484

**Published:** 2008-06-25

**Authors:** Hongying Jiang, Jigar J. Patel, Ming Yi, Jianbing Mu, Jinhui Ding, Robert Stephens, Roland A. Cooper, Michael T. Ferdig, Xin-zhuan Su

**Affiliations:** 1 Laboratory of Malaria and Vector Research, National Institute of Allergy and Infectious Diseases, National Institutes of Health, Bethesda, Maryland, United States of America; 2 Center for Global Health and Infectious Diseases, Department of Biological Sciences, University of Notre Dame, Notre Dame, Indiana, United States of America; 3 Advanced Biomedical Computing Center/NCI-Frederick, Frederick, Maryland, United States of America; 4 Bioinformatics Unit, Laboratory of Neurogenetics, National Institute on Aging, National Institutes of Health, Bethesda, Maryland, United States of America; 5 Department of Biological Sciences, Old Dominion University, Norfolk, Virginia, United States of America; Case Western Reserve University, United States of America

## Abstract

Mutations in PfCRT (*Plasmodium falciparum* chloroquine-resistant transporter), particularly the substitution at amino acid position 76, confer chloroquine (CQ) resistance in *P. falciparum*. Point mutations in the homolog of the mammalian multidrug resistance gene (*pfmdr1*) can also modulate the levels of CQ response. Moreover, parasites with the same *pfcrt* and *pfmdr1* alleles exhibit a wide range of drug sensitivity, suggesting that additional genes contribute to levels of CQ resistance (CQR). Reemergence of CQ sensitive parasites after cessation of CQ use indicates that changes in PfCRT are deleterious to the parasite. Some CQR parasites, however, persist in the field and grow well in culture, which may reflect adaptive changes in the parasite genome to compensate for the mutations in PfCRT. Using three isogenic clones that have different drug resistance profiles corresponding to unique mutations in the *pfcrt* gene (106/1^K76^, 106/1^76I^, and 106/^76I-352K^), we investigated changes in gene expression in these parasites grown with and without CQ. We also conducted hybridizations of genomic DNA to identify copy number (CN) changes in parasite genes. RNA transcript levels from 45 genes were significantly altered in one or both mutants relative to the parent line, 106/1^K76^. Most of the up-regulated genes are involved in invasion, cell growth and development, signal transduction, and transport activities. Of particular interest are genes encoding proteins involved in transport and/or regulation of cytoplasmic or compartmental pH such as the V-type H^+^ pumping pyrophosphatase 2 (PfVP2), Ca^2+^/H^+^ antiporter VCX1, a putative drug transporter and CN changes in *pfmdr1*. These changes may represent adaptations to altered functionality of PfCRT, a predicted member of drug/metabolite transporter superfamily found on the parasite food vacuole (FV) membrane. Further investigation of these genes may shed light on how the parasite compensates for functional changes accompanying drug resistance mutations in a gene coding for a membrane/drug transporter.

## Introduction

The identification of *pfcrt* as the key gene associated with chloroquine resistance (CQR) [1], [2] is one of the most important advances in our understanding of antimalarial drug resistance. Polymorphisms in the *pfcrt* gene were demonstrated to confer CQR using both drug selection and genetic transformation [1], [3], [4], [5], [6]. A lysine to threonine substitution at position 76 (K76T) has been found in every *in vitro*-tested CQR parasite from worldwide surveys of field isolates [7], [8]. However, the molecular mechanism of the PfCRT effect on parasite susceptibility to CQ remains controversial [9]. Furthermore, the endogenous role of PfCRT in the malaria parasite is not known. An understanding of the natural functions of PfCRT and other genes in the same cellular processes will provide a clearer picture of drug resistance evolution in the malaria parasite [10]. PfCRT is a 48 kDa protein with 10 predicted transmembrane-spanning domains and is located on the food vacuole (FV) membrane of intraerythrocytic stage parasites [1], [3]. Bioinformatic analysis suggested that PfCRT belongs to the drug-metabolite transporter (DMT) superfamily [11].

An unanswered question is whether the resistant *pfcrt* haplotype alone can generate a viable CQR parasite. Mutations conferring drug resistance often have deleterious effects on an organism 12, 13. Recent studies suggest that nucleotide substitutions in *pfcrt* may inflict some costs on parasite survival because discontinuation of CQ is sometimes followed by a slow reemergence of CQS parasites in the field [14], [15]. Conversely, some CQR parasites (e.g. the FCB and Dd2 lines) appear to be well adapted to growth *in vitro* even in the absence of drug pressure, suggesting potential compensatory or adaptive mutations that can overcome the deleterious changes in drug resistant genes. Compensatory mutations typically occur under drug selection to minimize the biological cost of acquiring the resistance mutation, and the resulting balanced genome minimizes the genetic burden of resistance [16]. The intense selection by CQ on *P. falciparum* has likely led to a cohort of alleles that may modulate the level of resistance and stabilize basic biological function of the cell. These additional mutations can be embedded in the same pathways and cellular processes as the gene conferring drug resistance or more changes in the same gene and may indirectly modulate the response level to the selecting drug.

*pfmdr1* has long been a focus of interest as a possible CQR gene, yet it remains unclear what role it plays in CQR [17]. One argument is that *pfmdr1* plays a compensatory role in CQR [18] while modulating responses to various drugs through indirect physiologic roles dependent on point mutations and copy numbers (CN) [19], [20], [21]. Because *pfmdr1* influences parasite responses to drugs with diverse structures and various modes of action, a non-specific mechanism may be invoked through changes in FV physiology. This hypothesis is supported by strong linkage disequilibrium between variants in *pfcrt* and *pfmdr1* [22], [23], perhaps involving a functional interplay between these proteins. Different clonal CQR parasite lines display a wide range of CQ IC_50_ values, indicative of incremental and additive effects on drug phenotypes even when the *pfcrt* and *pfmdr1* alleles are the same [23], [24]. The quantitative CQ response among the resistant isolates range from values 2.5 to 200-fold higher than their sensitive counterparts, even when *pfcrt* and *pfmdr1* are unchanged, indicating that the level of CQ response is influenced by additional genes [19], [23], [25].

CQR parasites have spread from as few as five origins to their current nearly global distribution, drastically altering the genomic landscape of malaria parasites [8]. While much effort has focused on understanding how key mutations in PfCRT produce CQR, strikingly little is known about the genetic background of CQR, the natural function of PfCRT and its molecular partners in the basic biology of the parasite.

Microarrays can be used to detect transcriptional changes on a genome basis and structural variation on the parasite chromosomes [26], [27], [28]. Studies have found that gene expression often evolves more rapidly than do coding regions of these genes [29]. These changes in gene expression can provide insights into the impact of acquired mutations, both random and adaptive, on transcription and drug resistance. To study parasite responses to mutations in *pfcrt* and the effects of these changes on parasite survival, we compared the genome-wide gene expressional profiles between two *pfcrt* mutant parasites derived by single-step drug selection from a CQS parasite line, 106/1^K76^ [3], [5]. We evaluated parasite gene expression in the presence and absence of sub-lethal CQ pressure and identified various genes with altered expression patterns, including those involved in growth, invasion and transport activities in the context of newly selected point mutations in PfCRT. We show that mutant parasites underwent simultaneous changes at multiple loci, including CN of *pfmdr1.*

## Result

### Global Gene Expression Profiles

To investigate parasite responses to mutations in the *pfcrt* gene, we hybridized total RNA extracted from parasites with different substitutions in the *pfcrt* gene. Raw data from 18 (six independent biological replicates for each parasite clone) array chips showed comparable data scales, distributions, and good RNA qualities with no apparent RNA degradation in any hybridizations (Figure S1, S2, and S3) [30]. The raw data were normalized to have the same quantile distributions; and genes with coefficient of variance (CV) ≥30% were filtered out. After normalization and processing, 3,356 (62%) reliable probe sets were retained for further analyses.

**Figure 1:**
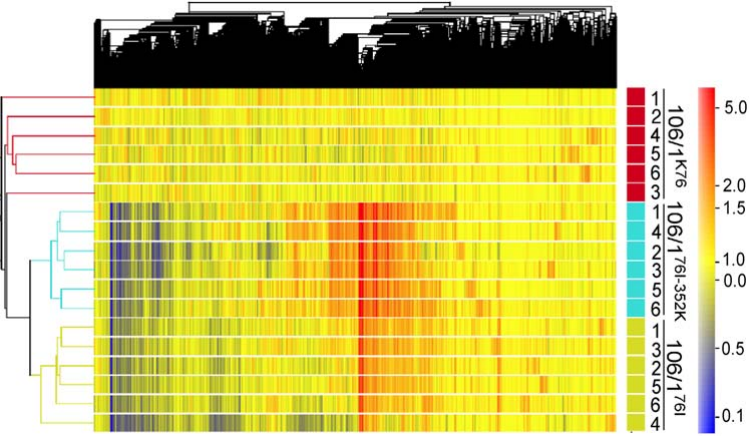
Hierarchal clustering of 3,356 filtered probesets for 18 samples/replicates. Each horizontal bar represents a sample or replicate; and each vertical line represents a probeset. Samples labeled in red are mRNA samples from 106/1^K76^; cyan are from 106/1^76I^; and yellow are from 106/1^76I-352K^. Replicates 1–3 were samples from parasites without CQ treatment; and 4–6 were those treated with CQ for 3 h. Gene expression levels were color-coded (color bar) from dark blue (log_2_ −0.01) to red (log_2_ 5).

To further evaluate the quality of our data and compare genome-wide gene expression profiles, we clustered all the 18 normalized microarray data sets using hierarchical clustering analysis (HCA). HCA showed that the majority of the 3,356 genes were not differentially expressed among all parasites (Figure 1) and that six replicates of each parasite had similar expression pattern. Additionally, 106/1^76I^ and 106/1^76I-352K^ showed similar up- and down-patterns and were clustered closer to each other than to their parent, 106/1^K76^. Each replicate of the same parasite line, both with and without CQ exposure, clustered closer to each other than to those from other parasites, indicating no significant CQ effect on global gene expression profiles.

Principle component analysis (PCA) on the whole genome expression data showed that the first two principal components (or two “super genes” derived from linear combination of expression data from of 3,356 genes) account for 82.8% and 7.7% of the variance in the data sets, respectively. Based on the first two components, there were three distinguished clusters consisting of six replicates from each parasite (Figure S4). The results suggested that these two components could be potentially used to find gene signatures or even used as signatures themselves to distinguish these parasites, similar to those observed in HCA. However, genes expressed at very low levels and with little variation among different treatments, although biologically important, could be missed in these analyses.

### Differentially Expressed Genes in the Mutant Parasites

Genes with 2-fold up- or down-regulation plus a false discovery rate (FDR) <0.001 were defined as significantly differentially expressed genes. Among the non-CQ treated parasites, when compared with the parental line 106/1^K76^, only 45 genes significantly changed expression in one or both mutant parasites that passed the stringent threshold (Table 1). Among the 34 up-regulated genes, 10 genes encoded proteins of unknown function. For the genes with putative functional assignment, genes encoding proteins associated with parasite invasion such as rhoptry proteins (4), merozoite membrane proteins (2), and apical membrane antigen (1) topped the list in both mutant parasites. In addition, 10 genes involved in parasite growth and cell cycle regulation, including two cysteine proteases (PFB0340c and PFB0345c), were also up-regulated.

**Table 1:** Genes significantly up- or down regulated in 106/1^76I^ and 106/1^76I-352K^ parasites

Gene name	106/1^K76^	106/1^76I^	106/1^76I-352K^	Annotation	Signal	TM
PFE0080c	0.99	7.01	40.13	rhoptry protein 2, RAP2	1–21	0
PF14_0102	1.00	8.02	32.97	rhoptry protein 1, RAP1	1–21	0
PFE0075c	0.98	4.95	28.18	rhoptry protein 3, RAP3	1–22	0
PFI0270w	0.99	5.47	20.53	intracellular trafficking	20–37	3
PFB0345c	1.00	5.75	18.11	cysteine protease	1–25	0
PFE0395c	0.99	6.04	14.23	hypothetical	1–21	1
MAL13P1.49	1.00	1.80	12.56	hypothetical	1–24	2
PFI0265c	1.00	4.44	11.76	rhoptry protein, RhopH3	1–24	0
PFE1130w	1.00	1.99	11.54	hypothetical	1–28	8
PFI1475w	1.00	2.43	7.82	MSP1	1–19	1
PFI1685w	0.99	3.62	7.62	cAMP-dependent kinase	0	0
PFL1385c	1.00	4.66	6.97	MSP9	1–23	0
PFL0260c	1.00	2.29	6.89	signal transducer	0	0
PF11_0373	1.00	1.36	6.81	cell cycle	1–18	0
PFE0340c	1.00	2.74	6.78	intracellular trafficking	0	6
PFE0895c	1.00	2.97	6.73	transcription regulator	0	0
PF11_0344	1.00	3.71	6.42	AMA1	1–24	1
PFB0340c	1.00	4.94	5.93	cysteine protease	0	0
PFD0240c	1.00	3.29	5.62	hypothetical	1–20	0
PFE0785c	1.00	2.44	5.29	drug/metabolite transporter	0	10
MAL13P1.130	1.00	2.40	4.75	hypothetical	0	6
PFB0845w	1.00	1.69	3.46	hypothetical	0	5
PFL1105c	1.00	1.48	3.13	hypothetical	0	1
MAL7P1.39	1.00	0.62	3.07	hypothetical	0	0
PFF0615c	1.00	1.28	3.07	protein pf12 precursor	1–25	1
MAL7P1.82	1.00	1.47	2.80	signal transducer	23–45	19
PF11_0066	1.00	1.50	2.74	caltractin/Ca2^+^-binding	0	1
PFF0170w	1.00	1.59	2.73	Ca2^+^/H^+^ antiporter VCX1	0	11
PFL1090w	1.00	1.76	2.71	vesicular transport	0	0
PFB0515w	1.00	0.93	2.59	oligosaccharide biosynthesis	1–29	4
PF11_0370	1.00	1.23	2.59	lipid transport	0	4
PFF0335c	1.00	1.47	2.49	hypothetical	1–20	0
PFL1080c	1.00	1.30	2.44	ATPase, PP-loop superfamily	0	0
PFF1180w	1.00	1.25	2.02	cell cycle control	0	0
PFE1115c	1.00	0.30	0.52	methyltransferase	0	0
PFL0700w	1.00	0.41	0.50	hypothetical	1–25	1
PFF0105w	1.00	0.64	0.47	MYND finger domain protein	0	0
PFF1430c	1.00	0.61	0.42	transport_amino acid	0	9
PF13_0219	1.00	0.55	0.38	hypothetical	0	0
PF14_0150	1.00	0.46	0.35	RNA polymerase	0	0
PF11_0265	1.00	0.52	0.26	vesicular transport	0	0
PF10_0016*	0.99	0.01	0.01	lipid transport	0	0
PF10_0019*	1.00	0.01	0.01	membrane protein	1–29	2
PF10_0020*	1.00	0.01	0.01	lipid transport	0	0
PF10_0021*	0.99	0.01	0.01	PHIST domain family	1–21	1

Values for 106/1^76I^ and 106/1^76I-352K^ represent significant fold changes (ANOVA; FDR<0.001) relative to 106/1^K76^ without CQ treatment. Fold change values represent means from three biological replicates. Signal, gene products with predicted signal peptide; TM, gene products with predicted transmembrane domains. ^*^ indicates deleted genes.

There were 11 genes that were significantly down-regulated or not expressed/deleted in one or both mutants (Table 2). Of interest, four of the genes (PF10_0016, PF10_0019, PF10_0020, and PF10_0021) reside within a ∼49 kb segment, including 3 contiguous genes at one end of chromosome 10, suggesting a chromosomal deletion. Two of the genes (PF10_0015 and PF10_0016) encode putative lipid transporters. The deleted genes were confirmed using real time PCR (data not shown) and DNA hybridization (see below).

Among the differentially expressed genes in the mutant parasites, at least six genes encode putative transporters (Table 1), three of which were involved in transporting Ca^2+^/H^+^ (PFF0170w), drug/metabolite (PFE0785c) and lipid (PF11_0370), respectively. For the down-regulated/deleted transporter genes, one was a putative amino acid transporter and two were lipid transporters. Relative to the 106/1^K76^ parasite, *pfcrt* was expressed 1.5-fold lower in both mutants; however, *pfmdr1* was 2-fold lower only in 106/1^76I^ (*pfmdr1* was not listed in Table 1 because it did not pass the FDR <0.001 test). As *pfcrt* is also a putative drug/metabolite transporter, changes in expression of various transporting molecules was probably not random, suggesting that mutations in PfCRT might disturb parasite transporting activities; and the parasites responded by altering expression of various transporters, particularly those transporting H^+^ and lipids. Of particular interest was the gene PFE0785c, as *pfcrt* was predicted to encode a drug/metabolite transporter too [11], [31].

In general, the patterns for both up- and down- regulated genes in the two mutant parasites were mostly co-linear or parallel (Table 1). However, for the up-regulated genes, the changes in gene expression in 106/1^76I-352K^ were generally greater than those in 106/1^76I^. The higher (or lower) levels of up- or down regulation of the genes could reflect parasite responses to additional change at PfCRT 352K and/or QN selection pressure.

### Expressional Changes under CQ Treatment

We also compared gene expression among the three parasites exposed to low dose (half dose of parasite IC_50_, see methods) CQ treatment for 3 h. We used the low drug concentrations because we sought evidence of a physiological response rather than the outright killing of parasites. Comparing gene expression of the same parasite with or without CQ treatments, no genes with significant changes (2 fold changes and FDR <0.001) in expression were found among all three parasites (two-way ANOVA) or between each parasite pair (t-test). HCA and PCA analyses clustered the samples from the same parasites with and without CQ treatments together, indicating minimum CQ effects on gene expression (Figure 1). We compared gene expression of the mutants with that of 106/1^K76^ after CQ treatment, and found 11 genes that significantly changed in the expression in one or both mutants, eight of which also changed expression without CQ treatment (Table 1 and 2). Of great interest was the 4 and 10 fold up-regulation of a gene encoding the vacuolar type H^+^ pumping pyrophosphatase 2 (PfVP2, PFL1700c) that belongs to a novel class of H^+^ pump molecules found in plants and many protozoa [32]. This protein could play a role in maintaining FV pH balance and in compensating mutations in PfCRT. Further investigation of the role of this gene and the gene encoding Ca^2+^/H^+^ antiporter in maintaining pH in the parasite and in CQR may lead to a better understanding of CQR and the biological functions of PfCRT.

**Table 2:** Genes significantly up- or down regulated in 106/1^76I^ and 106/1^76I-352K^ parasites with CQ treatment

Gene name	106/1^K76^	106/1^76I^	106/1^76I-352K^	Annotation	Signal	TM
PFE0080c	1.00	7.80	35.48	rhoptry protein 2	1–21	0
PFE0075c	0.92	8.65	33.77	rhoptry protein 3	1–22	0
PF14_0102	1.01	10.41	33.30	rhoptry protein 1	1–21	0
PFL1700c	0.97	4.05	10.15	V-type H^+^ pyrophosphatase	0	16
PFB0340c	0.99	4.90	6.20	cysteine protease	0	0
PFD0660w	1.00	1.49	2.12	phosphoglycerate mutase	0	1
PFC0080c	1.00	0.47	0.39	cell cycle control	20–38	2
PF10_0016	1.07*	0.02	0.02	lipid transport	0	0
PF10_0019	1.01*	0.01	0.01	membrane protein	1–29	2
PF10_0020	1.00*	0.01	0.01	lipid transport	1–21	1
PF10_0021	0.98*	0.01	0.01	hypothetical protein	0	0

Values for 106/1^76I^ and 106/1^76I-352K^ represent significant fold changes (ANOVA; FDR<0.001) relative to 106/1^K76^ with CQ treatment. Fold change values represent means from three biological replicates. Signal, gene products with predicted signal peptide; TM, gene products with predicted transmembrane domains. ^*^ indicates deleted genes.

### Functional Enrichment Analysis of Gene Ontology (GO) Biological Processes

**Figure 2:**
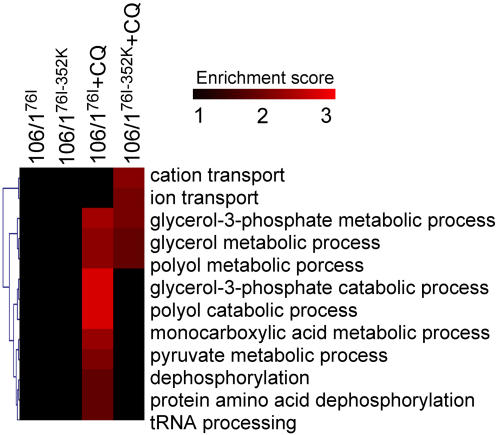
Gene Ontology (GO) biological processes enrichment levels among 2-fold differentially expressed genes in response to CQ treatment in both mutants. The enrichment levels (enrichment score) were measured by −log(P value of each term) from the Fisher exact test (see Methods). Red color indicates higher enrichment level. The analyses showed CQ-treated samples were highly enriched in glycerol and polyol metabolic processes in both mutant parasites.

Two-fold differently expressed genes from mutants exposed to CQ treatment were further analyzed for their biological pathways (GO terms). CQ-treated samples are highly enriched in glycerol and polyol metabolic processes in both mutant parasites (Figure 2). Enrichment of iron/cation transport activities was also found in the 106/1^76I-352K^ parasite, suggesting that CQ treatment could interfere with many relevant ion channels. These included genes encoding PfVP2, Ca^2+^/H^+^ antiporter, vacuolar ATP synthase (PF11_0412), and P-type ATPase (PFC0840w), although the last two genes did not pass our stringent criteria for selecting significantly differentially expressed genes (Figure S5). Mutations and/or over-expression of various transporters, especially transporters of the ABC superfamily, can confer drug resistance in organisms from bacteria to human cancer cell lines [33], [34]. Changes in PfCRT may affect parasite transport ability; and alteration in the activities of other transporters may be necessary for the parasite survival.

### Genes Expressed in Opposite Direction Between 106/1^76I^ and 106/1^76I-352K^

Because the two mutant parasites respond inversely to CQ and QN (106/1^76I^ is resistant to CQ, but sensitive to QN; 106/1^76I-352K^ is sensitive to CQ, but resistant to QN), we investigated if there were genes associated with the changes in drug responses. Surprisingly, there was no gene that was significantly (FDR = 0.01) up-regulated in one parasite but down-regulated in the other. PFI0115c was significantly expressed in reciprocal directions in the two mutants if the cut-off FDR was lowered to 0.05. PFI0115c encodes a protein kinase with 3.7 fold increased expression in 106/1^76I^, but 1.5 fold down in 106/1^76I-352K^. It is not clear what role this gene plays, particularly in response to CQ. The result supported the conclusion that opposite responses to CQ and QN in the two parasites were mainly due to mutations in PfCRT [5].

### Real Time RT-PCR Verification of Expressional Levels

**Figure 3:**
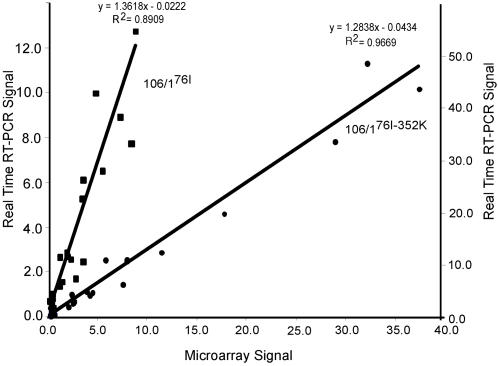
Real-time RT-PCR confirming some differentially expressed genes detected using microarray. Genes differentially expressed at various levels were selected for PCR verification. For the majority of the genes tested, the copy numbers of mRNA transcripts detected using real time RT-PCR correlated well with those of microarray (R^2^ were 0.8909 for 106/1^76I^ and 0.9669 for 106/1^76I-352K^). Squares represent 106/1^76I^; circles represent 106/1^76I-352K^.

To verify the expression changes seen in microarray hybridization, we used real-time RT-PCR to corroborate expression levels of selected genes. Primer sets were designed for each of 27 selected genes that were expressed at various levels among three parasites. Results showed that mRNA levels from real time RT-PCR correlated well with those from microarray (Figure 3). Three deleted genes (PF10_0016, PF10_0019, PF10_0020) showed almost no detectable signal in real-time PCR results. Pearson correlation coefficients of microarray expression and real-time RT-PCR data were 0.89 and 0.97 for 106/1^76I^ and 106/1^76I-352K^, respectively (excluding those deleted genes). PfVP2 (PFL1700c) had similarly increased expression levels in both CQ-treated and non-CQ-treated samples when estimated using real time RT-PCR. However, this gene was only listed in Table 2 with CQ treatment because it had CV >30% in non-CQ-treated samples and was filtered out during data preprocessing for the non-CQ-treated samples.

### Gene Deletion on Chromosome 10

**Figure 4:**
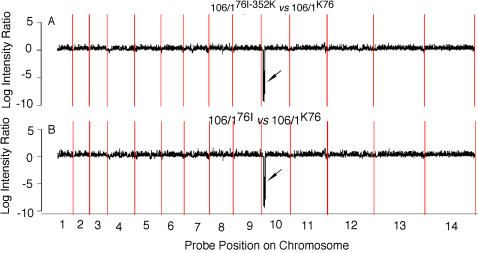
Gene deletions detected using DNA hybridization. Genomic DNA from 106/1^K76^, 106/1^76I^, and 106/1^76I-352K^ were hybridized to the same microarray. Hybridization signals of 106/1^76I-352K^ (A) or 106/1^76I^ (B) were subtracted from those of 106/1^K76^ after normalization and were plotted along each chromosome. X-axis represents the *P. falciparum* 14 chromosomes numbered 1 to 14; and Y-axes are the log ratio of signals from 106/1^76I-352K^ or 106/1^76I^ over that of 106/1^K76^, respectively. Note deletions of 10 genes at one end of chromosome 10 in both mutant parasites (arrows).

Hybridization of genomic DNA of the parasites to the same expressional array chip confirmed the deletion of 4 genes (Table 1) and an additional 6 genes (PF10_0011 to PF10_0025, chromosomal position 60068 to 108663) at the beginning of chromosome 10 as suggested in expression analyses (Figure 4 and Table S1). Actually, this region includes a total of 15 predicted genes. However, three of them (all hypothetical proteins) were not available on the chip. Among the genes deleted were genes encoding lipid transporters (PF10_0015 and PF10_0016), early transcribed membrane protein (PF10_0019), surface membrane proteins such as PfEMP1, and antigen PF70 (Table S1). These gene deletions were also confirmed using real time PCR (data not shown). Whether these genes play a role in parasite response to drugs will require further investigation.

### Parasite Growth and Invasion Rates

**Figure 5:**
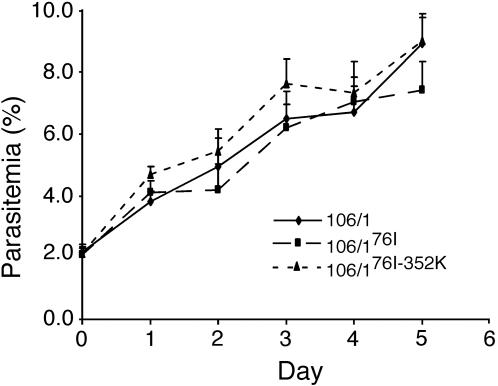
Parasite growth rates measured from *in vitro* culture. Parasitemias were obtained by daily counting of parasites from Geimsa-stained thin smears. Parasite cultures were started at 2% parasitemia and 2% haematocrit. Error bars are standard errors from 4–6 repeated measurements. The three parasite lines had similar growth rates in culture.

Many of the differentially expressed genes were putatively related to cell-cycle/growth or invasion. Therefore we investigated whether expression changes in these genes could affect parasite growth or invasion in culture. We compared the growth and invasion rates between the three parasites with the same starting parasitemia (2%) and haematocrit (2%). 106/1^K76^ and 106/1^76I-352K^ had very similar growth rates; but 106/1^76I^ growth rate was slightly lower (not significant) than the other two parasite lines (Figure 5). Lack of a significant decline in growth rates of drug-selected mutants is consistent with compensatory changes in the mutant parasites such as altered regulation of growth-associated genes, perhaps in response to deleterious effects of changes in *pfcrt*.

### The *pfmdr1* CN Variation, Expression Levels, and the Relationship to Drug Response

In addition to gene deletion on chromosome 10 and significant changes in expression of the genes listed in Table 1 and Table 2, changes in *pfmdr1* expression in the two mutant parasites (2-fold down-regulated in 106/1^76I^) were also detected. Changes in *pfmdr1* CN were associated with parasite responses to MQ and other drugs [20]. If we normalized *pfmdr1* CN to that of 106/1^76I^, 106/1^76I-352K^ and 106/1^K76^ had 2–5 copies, depending on methods used to estimate CN (Table 3). A higher CN of the *pfmdr1* gene and higher level of expression also appeared to correlate with higher IC_50_ to MQ and ART (Table 3). However, when we tested additional field isolates from Asia, the correlation disappeared (Table S2).

**Table 3:** *Pfmdr1* copy number (CN) estimates, mRNA expression levels, and parasite responses to four different drugs

Parasites	CN_RTPCR	CN_Array	Expr_RRPCR	Expr_Array	CQ	MQ	QN	ART
106/1^K76^	5.0	1.7*	3.3	2.5	11.1±0.2	5.0±0.3	84.9±3.5	11.1±1.2
106/1^76I-352K^	3.5	2.0	1.7	2.0	9.1±0.9	4.2±0.5	93.1±4.9	9.3±1.0
106/1^76I^	1.0	1.0	1.0	1.0	223.8±15.6	2.7±0.2	13.0±2.1	7.5±1.9

CN and mRNA levels were normalized to those of 106/1^76I^ (the lowest values). CN were averages of two different primer pairs using three different single copy genes (60S rRNA, β-tubulin, and PfEBA-175) as internal controls in PCR. Expression values from microarray were also averages of many separate experiments described in the text. Data for CQ (chloroquine), MQ (mefloquine), QN (quinine) and ART (artemisinin) represent IC_50_ values (nM) determined from 4–6 biological replicates. CN_RTPCR, relative CN determined using real time-PCR; CN_array, relative CN determined using microarray; Expr_RRPCR, relative mRNA level determined using real time RT-PCR; Expr_array, relative mRNA level determined using microarray.

* The CN was estimated to be 3 using a printed oligonucleotide array (data not shown).

## Discussion

Although PfCRT has been shown to play to a key role in CQR, the molecular mechanism of CQ action and CQR has not been resolved completely. Parasites resistant to CQ (and having mutations in PfCRT) accumulate less CQ, either due to a process of active energy-dependent CQ efflux [35], [36], [37] or passive efflux of diprotic CQ [38], [39], [40], a process that remains to be determined [9]. Importantly, the endogenous function and substrate(s) of PfCRT remain unknown. The inability to create *pfcrt* knockouts, its multi-transmembrane structure and cellular location suggest that PfCRT is involved in the transport of critical substrates into/out of the parasite FV. Several lines of experimental evidence indicated that mutations in PfCRT could affect FV membrane transport or pH balance; but how it exerts this activity is still being debated. Although controversial, parasites resistant to CQ were reported to have a lower pH in their FV than those in parasites with wild type PfCRT [41]. Therefore, in addition to affecting binding or transport of CQ, the biological functions of PfCRT may include maintaining pH balance in the FV or modulate the pH gradient across the FV membrane [42]. Heterologous expression in yeast (*Pichia pastoris*) and *Xenopus laevis* oocytes suggested that PfCRT had transporter activity that might affect membrane potential. Studies in *P. pastoris* revealed an increased proton (H^+^) gradient across the vesicular membrane with reduced pH inside, which was thought to be due to either the effect of PfCRT on chloride (Cl^−^) transport or ATPase activity [43]. Expression of PfCRT in *X. laevis* oocytes led to a reduced intracellular membrane potential and an alkaline pH relative to controls which were attributed to the activation of a non-selective cation transporter (Gcat) and an endogenous Na^+^-H^+^ exchanger [44]. However, expression of PfCRT in *Dictyostelium discoideum* resulted in only a small reduction in pH and significantly reduced verapamil-reversible intravesicular CQ accumulation in the mutant K76T lines relative to the control [37], suggesting a PfCRT-mediated energy-dependent efflux mechanism. Another possible role for PfCRT is the transport out the FV of amino acids resulting from hemoglobin digestion. Mutations in PfCRT may therefore affect these physiological processes.

This study identifies several putative transporters that might play a role in CQR or in adaptation to changes in PfCRT in *P. falciparum*. PfVP2, Ca2^+^/H^+^ antiporter VCX1, the putative drug transporter PFE0785c, and ATPase/synthase (PF11_0412 and PFC0840w) may represent molecules that the mutant parasites mobilize in response to the amino acid 76 mutation in PfCRT. Increased transcription of the gene encoding PfVP2 was seen in PfCRT mutant parasites with and without CQ pressure based on real-time RT-PCR. Although we did not see statistically significant changes in gene transcript levels between CQ-treated and non-treated, our GO functional enrichment analysis showed that CQ-treated mutants have significant enrichment in glycerol and polyol metabolic processes in both mutant parasites, and enrichment of iron/cation transport activities in the double mutant. This indicates that short-term CQ treatment may not significantly alter the expression of individual genes, but may assert small effects on groups of genes that together can cause a significant change at biological pathway/process levels.

One interesting gene with up-regulated transcripts in both *pfcrt* mutants was PFE0785c. This gene has 10 transmembrane domains and was recently annotated to encode a putative drug/metabolite transporter. PfCRT also has 10 transmembrane domains and was predicted to encode a putative drug/metabolite transporter [11]. The similarity between the two genes raises an interesting question on whether the two genes have similar functions in drug/metabolite transport. Three potential single nucleotide polymorphisms (SNPs) in coding regions and two in UTR regions of PFE0785c have been identified in PlasmoDB. Further investigation on the SNPs and parasite drug responses to different drugs may provide insight information on the function of this gene.

Two V type H^+^ pyrophosphatases (PfVP1 and PfVP2) have been described in *P. falciparum*, which constitute a novel class of H^+^ pump found in plants and some protozoa [32], [45]. The V-type H^+^ pyrophosphatases were found to be located on parasite plasma and vacuolar membranes, although the PfVP2 was expressed at a low level and could not be distinguished from PfVP1 [32], [45]. In experiments using inside-out plasma membrane vesicles, vesicles from yeast membrane carrying mutant PfCRT (Dd2 type) were found to have elevated ATPase and H^+^ transporting activities [43]. Because PfCRT does not have a recognizable ATPase domain, one of the potential explanations of this observation was that the mutant PfCRT might directly interact with H^+^-ATPase and become more efficient in stimulating both hydrolysis of ATP and H^+^ pumping in the presence of high symmetrical [Cl^−^] [43]. The up-regulation of the PfVP2 could be a response to the alteration in the ability of PfCRT to regulate FV pH. Nonetheless, these molecules appear to be present at the same location (FV membrane) and may work together in regulating the compartmental pH, which is closely related to accumulation of weak base drugs such as CQ. These results suggest that *pfcrt* may be indirectly involved in maintaining H^+^ balance in the parasite FV, consistent with previous reports of lower pH in CQR parasites with mutant *pfcrt* [41]. Further investigation of the roles of PfVP2 and the gene encoding Ca^2+^/H^+^ antiporter in maintaining pH in the parasite and in CQR may lead to a better understanding of CQR in *P. falciparum* and the biological functions of PfCRT.

Another interesting observation was the change in *pfmdr1* CN accompanying the CQ-selected *pfcrt* mutations. *pfmdr1* CN decreased from 5 copies to 1 copy after CQ selection of 76I (from the original 76K) in PfCRT; and increased to ∼4 copies after QN selection (real time PCR data). mRNA levels were similarly changed in response to *pfcrt* mutations and drug selection (Table 3) and support the argument CN changes influence *pfmdr1* expression in response to CQ selection and/or mutations in PfCRT. Increased CN of *pfmdr1* have also been associated with increased IC_50_ values to MQ both in field isolates and laboratory-selected parasites [20], [46]. Interestingly, MQ-selected parasites also showed an inverse relationship between CQ response levels and increased *pfmdr1* gene CN [46]. The proteins encoded by *pfcrt* and *pfmdr1* are located at the FV membrane [3], [47], suggesting a contribution to the FV function and potential direct interactions. Because our *pfcrt* mutants were selected using lethal doses of CQ or QN to provoke the emergence of pre-existing mutants, it is possible that *pfmdr1* CN variants were also present before drug selection, suggesting that changes in *pfmdr1* CN might partner with specific *pfcrt* alleles. Substitutions in amino acids of PfMDR1 (Pgh1) could be another way that the parasite compensate for the *pfcrt* mutations as certain *pfmdr1* alleles have been shown to be closely linked to different mutant *pfcrt* alleles [22], [23], [48]; however, no substitution in the *pfdmr1* was observed among the two mutant parasites [5]. Increased *pfmdr1* CN also appeared to have higher mRNA expression levels and slightly higher IC_50_ for MQ/ART among the three parasite lines (Table 3), which were not selected with MQ or ART. The results also suggest that the observed association of higher MQ IC_50_ and *pfmdr1* CN in field parasites may be a combined result from selection of multiple drugs such CQ, QN, and MQ. However, *pfmdr1* CN change is unlikely to be the major determinant causing changes in IC_50_ for the drugs we tested as there were no clear correlation between *pfmdr1* CN and CQ IC_50_ (Table S2). Given the opposite selection pressure of CQ and MQ/ART on *pfmdr1*, addition of CQ to ART treatment may improve the efficacy of ART, although CQ alone is not effective in many parts of the world.

Mutations in PfCRT may affect the parasite growth or survival. There is evidence showing that parasites with mutations in PfCRT may have reduced ability to compete with wild type parasites. In some endemic regions of Africa and Asia, cessation of CQ use has resulted in the return of parasites with wild type PfCRT among parasite populations highly resistant to CQ [14], [15], [49]. Additionally, most of the field PfCRT mutant parasites carry multiple amino acid substitutions. Simultaneous changes of multiple positions in PfCRT suggest that the substitution at amino acid position 76 may also require changes at other positions in the molecule in order to maintain cellular homeostasis [1], [8], although such changes may not restore full activity of PfCRT. The requirement of other substitutions to compensate for changes at amino acid position 76 can explain why PfCRT 76 mutations could only be selected from parasite 106/1^K76^ in *in vitro* culture that has several pre-existing mutations in the molecule, but not from other wild type parasites [3], [5]. The majority of the individual changes in PfCRT probably result in a fitness cost for the parasite, explaining why only one or two amino acid changes in PfCRT have not been observed in laboratories or in the field populations. Requirement of multiple changes simultaneously in PfCRT can also explain relatively rare events of founder mutations for CQR [50]. Therefore, compensatory changes in the genetic background of the key position 76 PfCRT CQR mutation could occur at several levels. The first one is changes within PfCRT itself. The substitution at position 76 is critical for conferring CQR, and other changes that vary with independent evolutionary origins of CQR can be directly involved in the CQR mechanism or can be simply compensating biological function of the protein. Compensatory changes within PfCRT may not fully restore the biological functions of the protein, thereby requiring further changes in other parts of the genome, presumably influencing expression or function of genes whose functional products relate to the fitness impacts of the natural biology of PfCRT. Notably, the expressional changes we report are limited to potential compensatory changes in other genes in response to substitution of 76I or 325K in *pfcrt*.

Compensatory changes for drug resistant mutations have been widely reported in microorganisms. One example is the compensatory adaptation to the loss of fitness associated with changes in elongation factor G conferring resistance to fusidic acid in *Staphylococcus aureus* [13]. In our study, many genes highly up-regulated in RNA expression were those involved in parasite invasion (rhoptry proteins and merozoite surface proteins) and growth (proteins in cell cycle, transcription and translation regulators, and proteases). In malaria parasites, cysteine proteases have been shown to play a role in merozoite release from the erythrocyte [51]. Our results showed that the two *pfcrt* mutants had significant increase in transcription levels of many genes that were related to parasite invasion and growth, although some of the responses might be non-specific to the two *pfcrt* mutations. However, the two PfCRT mutants had similar growth and invasion rates as the parent line 106/1^K76^ in regular erythrocyte culture (Fig. 5). One possibility is that despite high transcription levels, protein expression of these genes may change minimally. Other possibilities are redundancy of parasite mechanisms related to growth and invasion, or the changes in expression of these genes were responses to deleterious impacts on growth.

The genes deleted on chromosome 10 included four genes encoding antigen/parasite membrane (e.g. PfEMP1, PF70), two encoding acyl CoA binding protein or lipid transporters, two genes encoding alpha/beta hydrolases, the rest two genes was recently assigned to *Plasmodium* helical interspersed subtelomeric (PHIST) family and contain a PEXEL trafficking motif. Interestingly, in the middle of those deleted genes, PF10_0014 did not show much difference in hybridization signals among these 3 parasites. The functional effects of these deletions on parasite growth and response to CQ or PfCRT mutations are unknown.

In summary, mutations in PfCRT caused limited changes in *P. falciparum* global RNA gene expression with or without short-term CQ pressure, even though parasite sensitivity to CQ and QN were dramatically altered. Therefore, the changes in CQ and QN resistance phenotypes in the mutants could be largely attributed to the key mutation(s) in PfCRT. However, mutations in PfCRT, particularly at amino acid position 76, could affect normal cellular functions, possibly in maintaining FV pH. The loss (or reduction) of function in PfCRT could be compensated through increased expression of some other transporters such as PfVP2 and/or changes in CN of *pfmdr1*. Other compensatory changes not detected by this analysis might have also occurred; and the changes identified in this study will require further investigations that may provide insights into the endogenous function of PfCRT and its role in CQR in *P. falciparum*.

## Materials and Methods

### *P. falciparum* Parasites and *in vitro* Culture

Parasites were cultured *in vitro* according to the methods of Trager and Jensen [52]. Briefly, parasites were maintained in RPMI 1640 medium containing 5% human O^+^ erythrocytes (5% haematocrit), 0.5% Albumax (GIBCO, Life Technologies, Grand Island, NY), 24 mM sodium bicarbonate and 10 μg/ml gentamycin at 37°C with 5% CO_2_, 5% O_2_, and 90% N_2_. *P. falciparum* isolate 106/1^K76^ and its two isogenic PfCRT mutants 106^76I^ and 106^76I-352K^ were used in this study [5]. Parasite 106/1^K76^ has six mutations found typically in Southeast Asian CQR parasite (compared with 3D7 and other CQS wild type parasites), except a key mutation at PfCRT 76 position [1]. 106/1^K76^ is sensitive to CQ (IC_50_ = 34 nM), but relatively resistant to QN (IC_50_ = 205 nM). The mutant parasite 106^76I^ was derived from 106/1^K76^ using a lethal dose of CQ (100 nM) that selects for a pre-existing parasite with mutation at 76 position. 106^76I^ is CQR (IC_50_ = 589 nM) but exceptionally sensitive to QN (IC_50_ = 16 nM) [3]. 106/1^76I-352K^ was derived from 106/1^76I^ using a lethal dose of QN (100 nM); and the parasite reverted to CQS (IC_50_ = 26 nM) and became more resistant to QN (IC_50_ = 240 nM) [5]. The identity of each parasite clone was confirmed using microsatellite genotyping and sequencing the entire *pfcrt* open reading frame [53].

### RNA and DNA Extraction

Parasites were synchronized in three successive steps: 1) 5% sorbitol treatment of ring stage, 2) Percoll-sorbitol (80%∶40%) gradient purification of schizonts after 24 h, and 3) another round of 5% sorbitol treatment of ring after another 10–12 h [54], [55]. Total RNA was isolated 24 h after the last step of synchronization with parasites (∼5%) mostly at trophozoite stage in 150 ml flasks (Figure S6) using Trizol extraction protocol according to the manufacturer's instruction (Invitrogen, Carlsbad, CA). We used trophozoite because this is the stage that is actively growing and is sensitive to many drugs, including CQ [56]. All RNA samples for each parasite and each treatment were prepared at different times (three biological replicates). The quantity and quality of the RNA samples were estimated using spectrophotometry, agarose gel electrophoresis, and Agilent 2100 Bioanalyzer (Foster City, CA). Genomic DNA was extracted from saponin-lysed parasite pellets using Wizard Genomic DNA Purification Kit (Promega, Madison, WI).

### CQ Treatment

For testing parasite expressional changes in response to short-term low dosage of CQ, we cultured parasites with or without CQ for 3 h before RNA extraction based on previously established IC_50_ levels of each parasite [5]. The CQ (chloroquine diphosphate salt, Sigma-Aldrich, St. Louis, MO) concentrations used were half of the IC_50_ value for each parasite: 106/1^K76^, 15 nM; 106/1^76I^, 295 nM; 106/1^76I-352K^, 10 nM [4].

### Invasion and Growth Assays

Schizonts were purified using percol/sorbitol gradient (80%∶40%) and diluted to 2% initial parasitemia in 2% haematocrit. For invasion rate, the numbers of ring stage parasites from 10–20 microscope fields (∼2000 RBCs) were counted as described [57] after 18–20 h of culture. The parasites were also stained with EtBr and counted using a flow cytometry after 48 h of culture. Growth rates were monitored everyday after synchronization by counting the parasitemia using the above two methods. Culture media were changed everyday, and the assays were repeated 4–6 times on all parasites.

### RNA and DNA Labeling and Hybridization

The Johns Hopkins *Plasmodium/Anopheles* Expression Array (http://www.affymetrix.com/products/arrays/specific/plasmodium.affx) that has 5,407 probsets covering 4,700 predicted *P. falciparum* genes were purchased from Affymetrix (Santa Clara, CA). The array contained short oligonucleotides (25-mer) synthesized *in situ* on a glass slide, with each predicted coding region having multiple probes (11 probe pairs per gene). RNA labeling (5 μg total RNA) and hybridization were performed following Affymetrix standard protocols for eukaryotes using One Cycle Target Labeling and Control Reagent Kit. Genomic DNA was also fragmented to an average size of 50–150 bp with DNase I and subsequently end-labeled using terminal deoxynucleotidyl transferase (TdT) and a biotin labeling kit (Affymetrix Mapping 250K reagent kit). Biotin-labeled cRNA and cDNA were hybridized to the chip at 45°C for 16 h with constant rotation at 60 RPM. Affymetrix 20X hybridization control was used to make the hybridization cocktail.

### Microarray Signal Scanning and Data Analysis

Hybridized chips were washed and stained following the company's EukGE-WS2v5 protocol. The chips were scanned at 570 nm emission wavelength using Affymetrix scanner 3000. Image CEL files were processed and normalized using the Robust Multi-array Analysis with correction for GC content of the oligos (GC-RMA) [58], and the processed data were imported into GeneSpring (v7.2, Agilent Technologies) for further data filtering, normalization, and analyses. To maximize the proportion of high quality transcripts, data were filtered using a coefficient of variance (CV) cut-off value of 30%. Signal from each gene was normalized to the means of three 106/1^K76^ replicates (non CQ-treated). Default settings in GeneSpring were used for hierarchical clustering and principal component analysis (PCA) for all the samples. Differentially expressed genes were identified based on 2-fold up- or down changes and significance at FDR of 0.001 (Benjamini and Hochberg FDR multiple test correction) using analysis of variance (ANOVA). Functional annotation of *P. falciparum* genes was based on those in PlasmoDB and additional Blast searches of various databases. All raw data and minimal information about a microarray (“MIAME”) checklist were submitted to NCBI Gene Expression Ominibus (http://www.ncbi.nlm.nih.gov/geo/query/acc.cgiaccGSE10022) and PlasmoDB (http://www.plasmodb.org/plasmo/home.jsp).

### Functional Enrichment Analysis Using Gene Ontology

The functional enrichment analysis was performed using WPS software [59]. Genes associated with biological processes, molecular functions and cellular components were derived from Gene Ontology (GO) Consortium (http://www.geneontology.org) and were used for functional enrichment analysis. Briefly, the one-sided Fisher's exact test based on 2×2 contingency tables (whether a gene is in a given list or not *vs.* whether this gene is associated with a GO term or not) was used to determine which GO term had a statistically significant enrichment within differentially expressed genes. The permutated *P* value or FDR for pathway enrichment was calculated with a permutation number of 300. Results of multiple gene lists from the Fisher exact test were compiled and displayed in color-coded heat maps to reveal the patterns of significantly altered biological processes and pathways. The color-coding of the heat maps is based on the enrichment score of genes in a pathway (−Log (*P* value) of each term) with red color indicating a higher level of enrichment.

### Real Time Quantitative RT-PCR

Real time RT-PCR was performed to verify expressional levels of selected genes; and real time PCR was used to determine CN of *pfmdr1* in the parasites. First strand cDNA synthesis was performed using Superscript First Strand Synthesis System kit (Invitrogen) and oligo-dT and random hexamers as primers. Quantitative real-time PCR was performed in an iCycler iQ™ multicolor real-time detection system (Bio-Rad) using the iQ SYBR Green Supermix (Bio-Rad).

Primers for real-time PCR (Table S3) were designed to produce amplicons of 80–150 bp after blasting against the whole genome to ensure specificity. A total of 27 genes with different levels of expression detected using microarray were selected for real-time PCR verification using the same RNA purified for microarray hybridizations. For estimating the CN of *pfmdr1*, we used two sets of primers. One pair of primers was the same as those described [20]. The sequences of second pair of primers were: forward 5′-ACTATTGCCCACAGAATTGC-3′ and reverse 5′-CCATCTTGTGCTGATAATAATTC-3′. The quantity of cDNA/DNA was first calibrated using serial-diluted plasmid DNA, then normalized using internal control genes encoding 60S ribosomal subunit protein L18 (MAL13P1.209) for RNA, and β-tubulin (PF10-0084) and PfEBA175 (MAL7P1.176) for gDNA, and finally expressed as relative levels to the parent line 106/1^K76^ in each set of experiment. The reactions were carried out according to manufacturer's instructions with the following cycling program: 5 min at 95°C for initial denaturation; 95°C for 30 s, 50°C for 30 s, 60°C for 30 s for 45 cycles; and a final extension at 55°C for 1 min.

### Antimalarial Drug Assay

Drug assays were performed using SYBR Green I method modified from a procedure described previously [60]. Briefly, parasites were diluted to 1% parasitemia in 1% haematocrit and aliquoted (150 μl) into a 96-well plate with wells containing 50 μl of a serial-diluted drugs or drug-free controls. After incubation at 37°C for 72 h, DNA were released from cultured parasites and stained with SYBR Green I dye as described. The plate was kept in dark for 30 min and signals were read in a FluoStar microplate reader (BMG Labtech, Germany). IC_50_ values were calculated using nonlinear regression to yield the drug concentration that kills 50% of the parasites relative to the drug-free controls. Data were fitted to a sigmoidal dose-response/variable slope equation using the GraphPad Prism 5.0 software package (San Diego, CA).

## Supporting Information

Figure S1 Boxplots of unprocessed log scale probe intensities. Samples in red were from 106/1K76; in blue were from 106/176I; and in yellow were from 106/176I-352K. Among the six samples, three were treated with CQ and other three were untreated
(0.08 MB TIF)

Figure S2 Distribution of probeset intensity. Each line represents one of the 18 microarray chips tested. Plotted on the Y-axis is mean intensity by probeset position. Intensities have been shifted from original data for better viewing (due to overlapping lines), but slopes are unchanged.
(0.11 MB TIF)

Figure S3 MA-plots showing distribution of normalized data. Each comparison consists of averaged data from six chips representing each of the three parasites. M is the log-ratio of the expression intensities between two parasites, whereas A is the mean log-expression intensities between two parasites. The red lines are the LOESS smoother/regression lines.
(0.15 MB TIF)

Figure S4 Principal component analysis of whole genome expression data (3,356 probesets/genes) from 18 hybridizations. The first two principal components account for 82.8% and 7.7% of the variance in the data sets, respectively. Each dot or circle represents one hybridization. Dots in red are samples treated with chloroquine.
(0.21 MB TIF)

Figure S5 Up-regulated expression of genes associated with iron/cation transport in the PfCRT mutants. Each segment of the gene bars represents expressional levels (as color-coded) of the genes in each parasite (represented as a segment); and the levels of expression in fold changes are as labeled. The percentages under/above the four segments of the GO term bars (iron and cation transport) indicate the percentage of annotated genes for the intended GO term from the corresponding dataset reaching the color-coded criteria. For example, 12% of the genes belonging to cation transport were 2-fold up-regulated in the 106/176I-352K parasite after treated with CQ. The thin lines indicate each of the two GO terms and their associated genes.
(0.19 MB TIF)

Figure S6 Micrograph showing synchronized trophozoites prior to RNA extraction. Parasites were stained with 1% Giemsa. A, 106/1K76; B, 106/176I; C, 106/176I-352K
(7.91 MB TIF)

Table S1 Genes deleted in 106/176I or 106/176I-352K parasites
(0.01 MB XLS)

Table S2 pfmdr1 copy numbers and parasite responses (IC50, nM) to antimalarial drugs. CN was estimated using real time PCR described in the text. The additional parasite isolates were described previously [21]. CN, copy number; CQ, chloroquine; MQ, mefloquine; QN, quinine, and ART, artemisinin. IC50 values were determined using 4–6 independent replicates. Copy numbers were adjusted to one copy for 106/176I.
(0.04 MB DOC)

Table S3 Primer sequences used in real time PCR
(0.02 MB DOC)
