# Supplementary figures and images for: Genome-Wide Compensatory Changes Accompany Drug- Selected Mutations in the Plasmodium falciparum crt Gene
Source: PLoS One. 2008 Jun 25;3(6):e2484. doi: 10.1371/journal.pone.0002484 (PMC2424241; doi:10.1371/journal.pone.0002484)

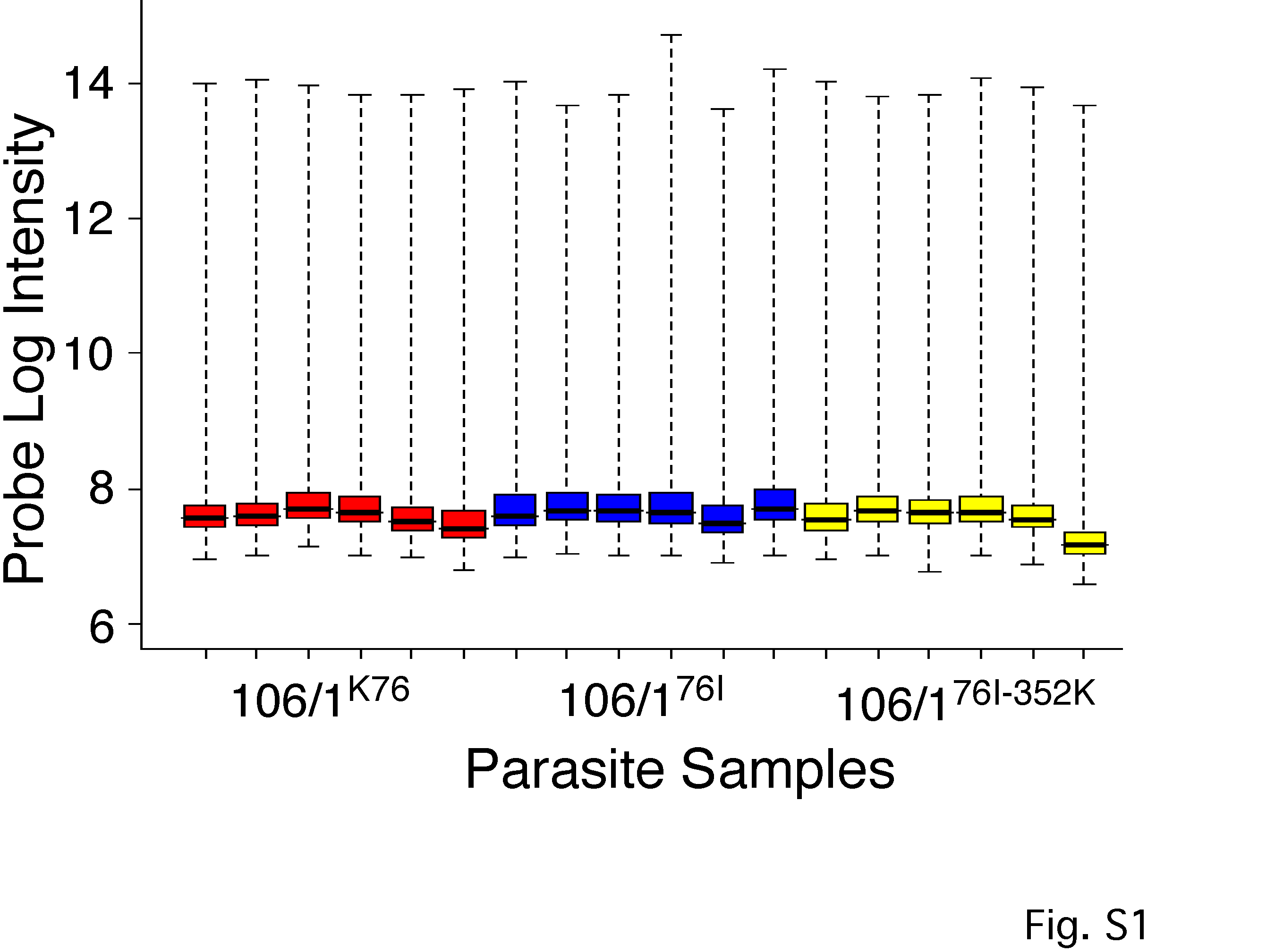

Supplement: Figure S1 — Boxplots of unprocessed log scale probe intensities. Samples in red were from 106/1K76; in blue were from 106/176I; and in yellow were from 106/176I-352K. Among the six samples, three were treated with CQ and other three were untreated (0.08 MB TIF) [file pone.0002484.s001.tif]

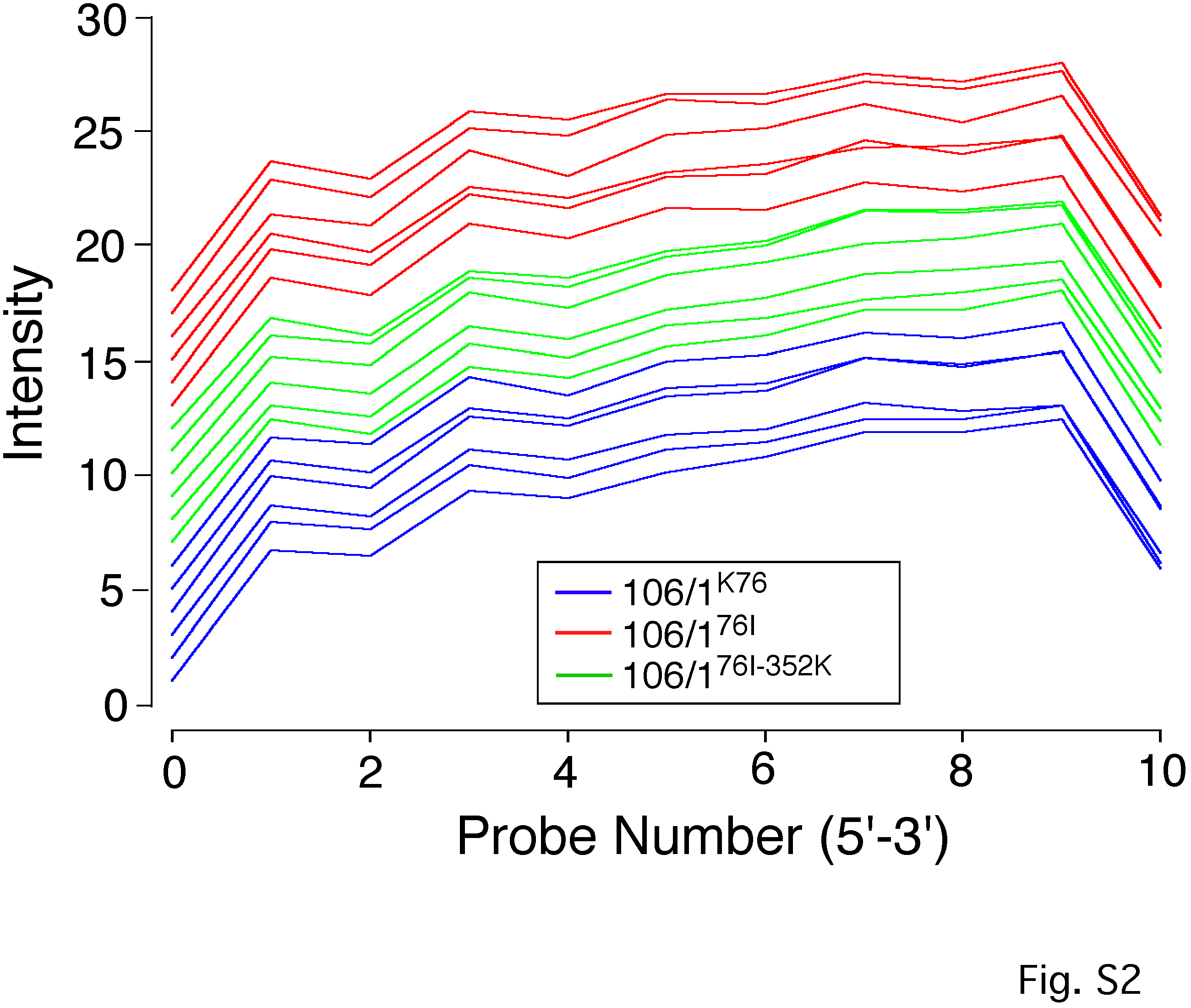

Supplement: Figure S2 — Distribution of probeset intensity. Each line represents one of the 18 microarray chips tested. Plotted on the Y-axis is mean intensity by probeset position. Intensities have been shifted from original data for better viewing (due to overlapping lines), but slopes are unchanged. (0.11 MB TIF) [file pone.0002484.s002.tif]

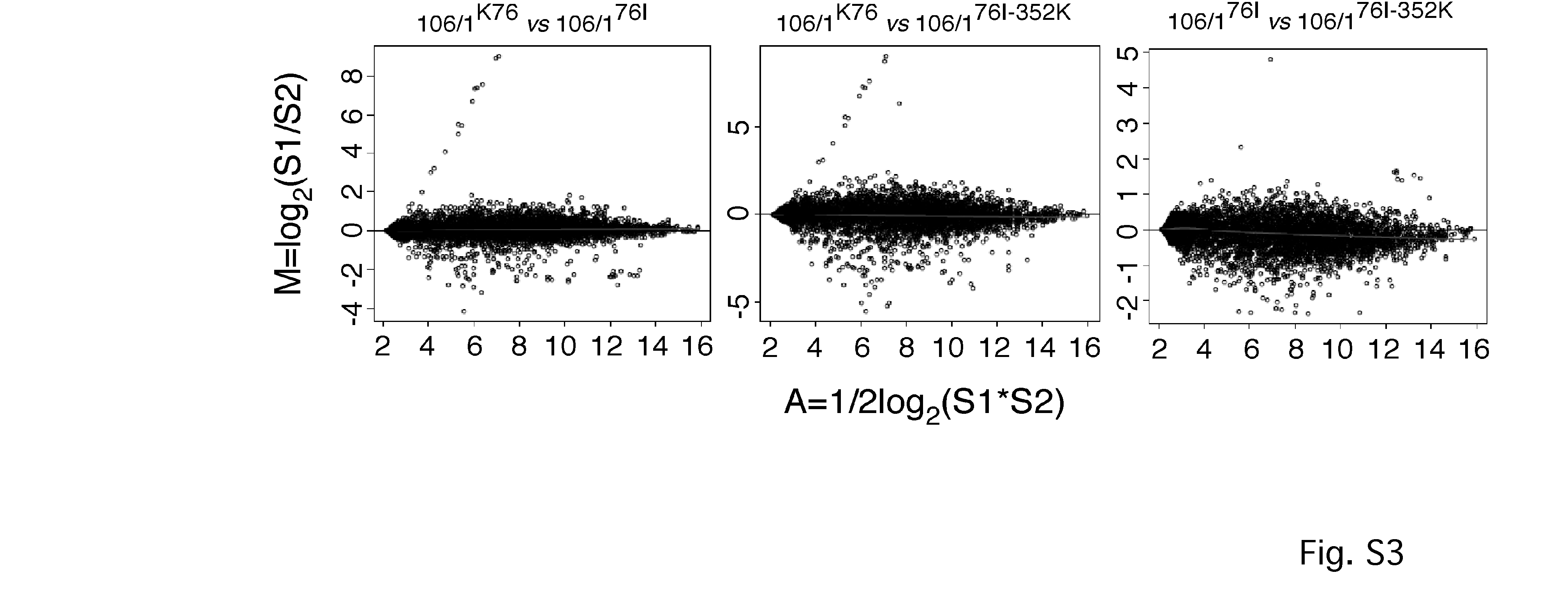

Supplement: Figure S3 — MA-plots showing distribution of normalized data. Each comparison consists of averaged data from six chips representing each of the three parasites. M is the log-ratio of the expression intensities between two parasites, whereas A is the mean log-expression intensities between two parasites. The red lines are the LOESS smoother/regression lines. (0.15 MB TIF) [file pone.0002484.s003.tif]

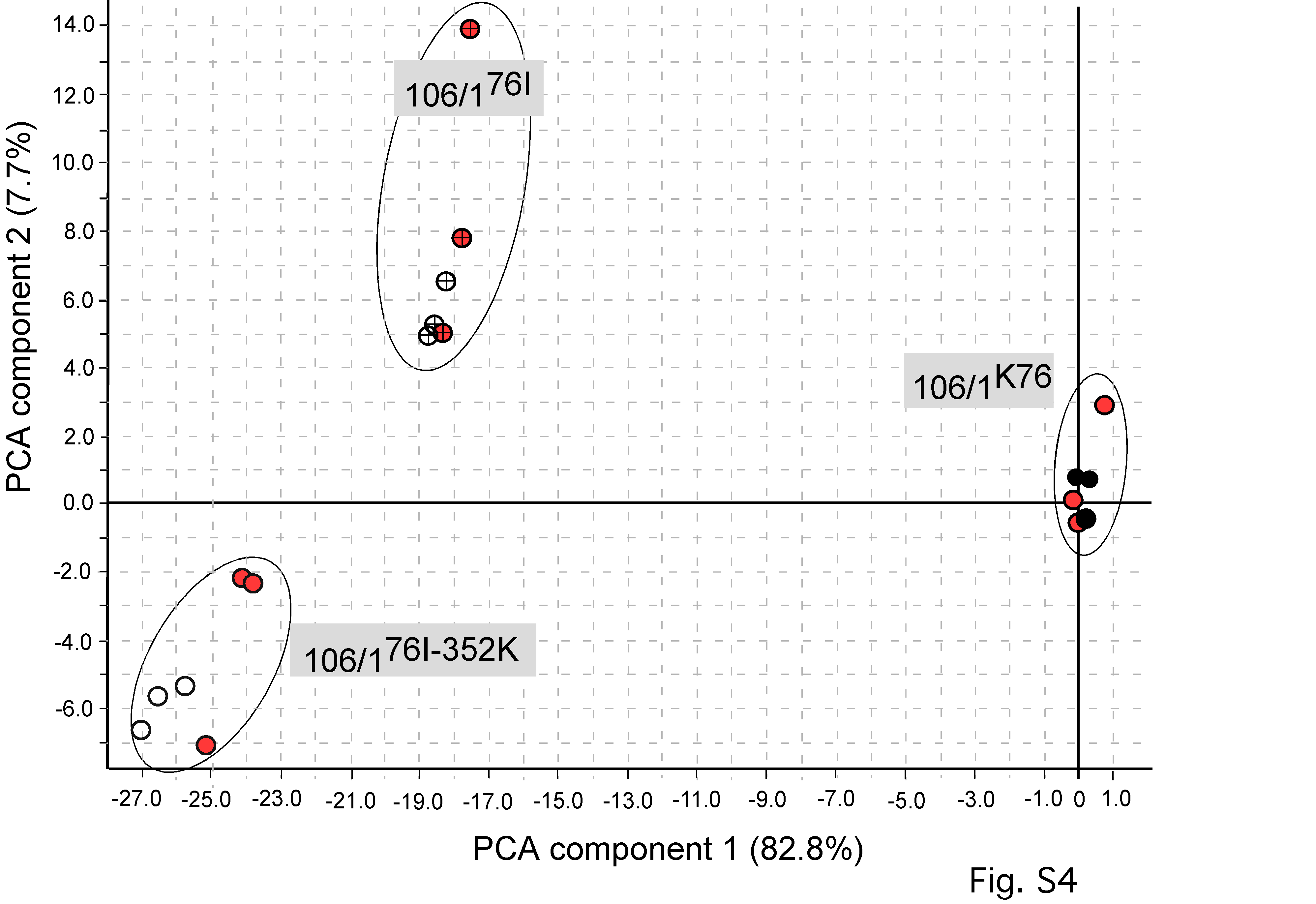

Supplement: Figure S4 — Principal component analysis of whole genome expression data (3,356 probesets/genes) from 18 hybridizations. The first two principal components account for 82.8% and 7.7% of the variance in the data sets, respectively. Each dot or circle represents one hybridization. Dots in red are samples treated with chloroquine. (0.21 MB TIF) [file pone.0002484.s004.tif]

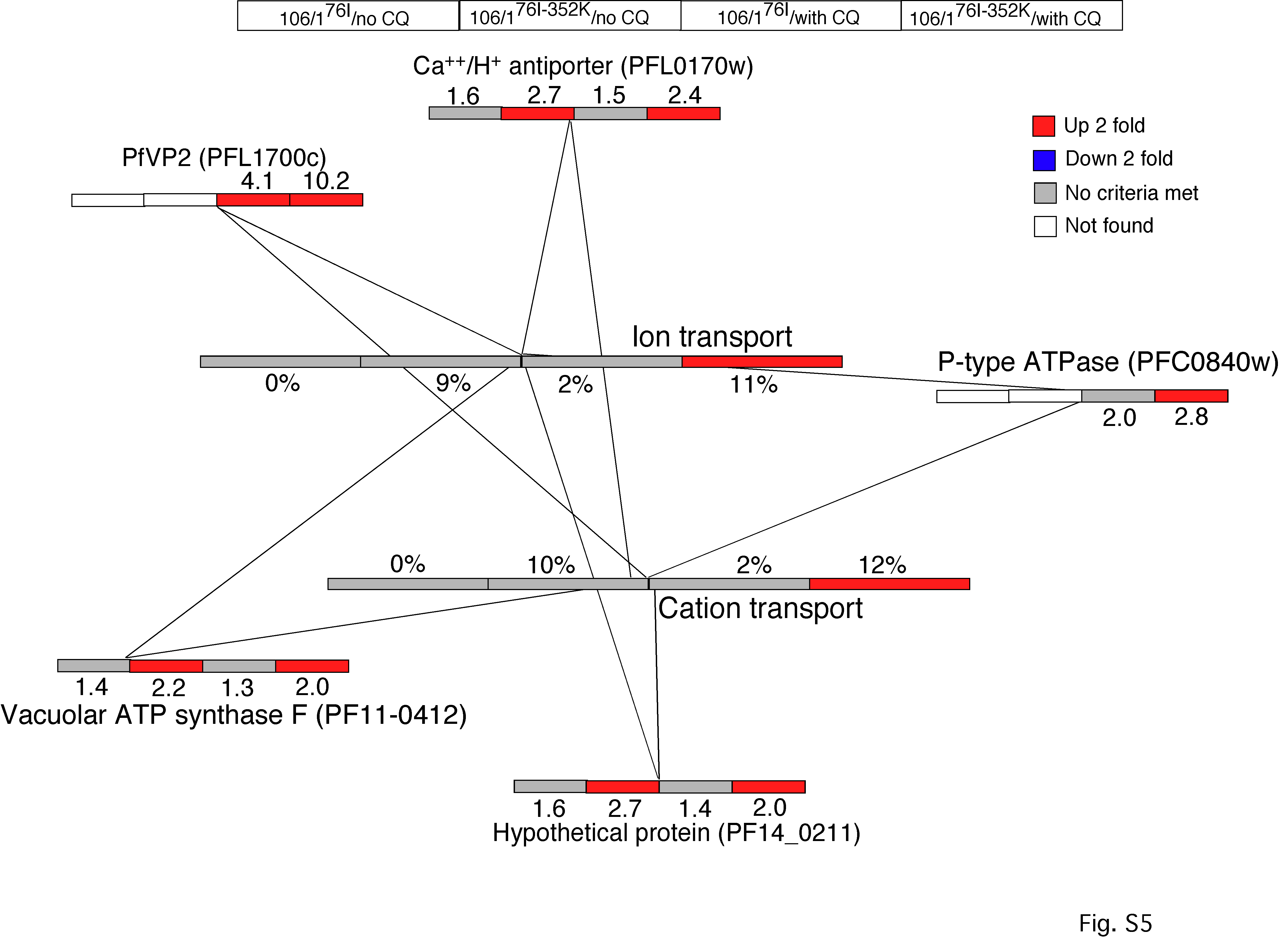

Supplement: Figure S5 — Up-regulated expression of genes associated with iron/cation transport in the PfCRT mutants. Each segment of the gene bars represents expressional levels (as color-coded) of the genes in each parasite (represented as a segment); and the levels of expression in fold changes are as labeled. The percentages under/above the four segments of the GO term bars (iron and cation transport) indicate the percentage of annotated genes for the intended GO term from the corresponding dataset reaching the color-coded criteria. For example, 12% of the genes belonging to cation transport were 2-fold up-regulated in the 106/176I-352K parasite after treated with CQ. The thin lines indicate each of the two GO terms and their associated genes. (0.19 MB TIF) [file pone.0002484.s005.tif]

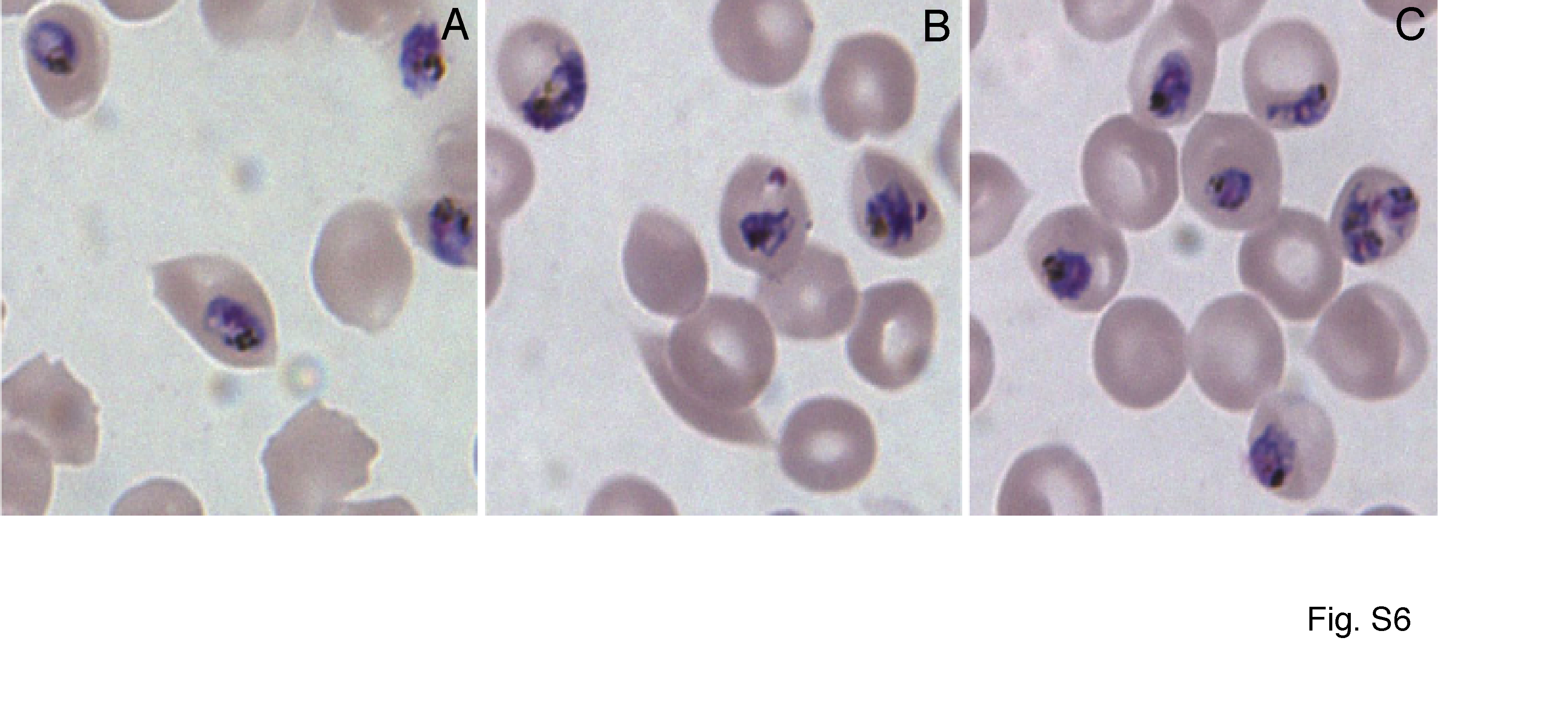

Supplement: Figure S6 — Micrograph showing synchronized trophozoites prior to RNA extraction. Parasites were stained with 1% Giemsa. A, 106/1K76; B, 106/176I; C, 106/176I-352K (7.91 MB TIF) [file pone.0002484.s006.tif]
