# Supplementary material for: Genome-Wide Compensatory Changes Accompany Drug- Selected Mutations in the Plasmodium falciparum crt Gene
Source: PLoS One. 2008 Jun 25;3(6):e2484. doi: 10.1371/journal.pone.0002484 (PMC2424241; doi:10.1371/journal.pone.0002484)
Supplement: Table S2 — pfmdr1 copy numbers and parasite responses (IC50, nM) to antimalarial drugs. CN was estimated using real time PCR described in the text. The additional parasite isolates were described previously [21]. CN, copy number; CQ, chloroquine; MQ, mefloquine; QN, quinine, and ART, artemisinin. IC50 values were determined using 4–6 independent replicates. Copy numbers were adjusted to one copy for 106/176I. (0.04 MB DOC) [file pone.0002484.s008.doc]

**Table S2.** *pfmdr1* copy numbers and parasite responses (IC_50_, nM)

to antimalarial drugs

Parasites CN CQ MQ QN ART

Dd2 3.4 177.0 5.9 117.0 10.9

106/1^76k^ 2.8 9.8 5.0 84.9 11.1

C2A 2.7 123.0 28.2 289.0 31.0

106/1^76I-352K^ 2.0 9.1 4.2 93.1 9.3

98-5 1.2 219.0 23.1 171.1 13.2

3D7 1.0 5.2 12.7 30.9 21.4

Thai2 0.9 50.3 30.2 186.0 11.1

106/1^76I^ 0.5 188.0 2.7 13.0 7.5

CN was estimated using real time PCR described in the text. The additional parasite isolates were described previously [Mu, 2003 #1864]. CN, copy number; CQ, chloroquine; MQ, mefloquine; QN, quinine, and ART, artemisinin. IC_50_ values were determined using 4-6 independent replicates.
