# Supplementary material for: Genome-Wide Compensatory Changes Accompany Drug- Selected Mutations in the Plasmodium falciparum crt Gene
Source: PLoS One. 2008 Jun 25;3(6):e2484. doi: 10.1371/journal.pone.0002484 (PMC2424241; doi:10.1371/journal.pone.0002484)
Supplement: Table S3 — Primer sequences used in real time PCR (0.02 MB DOC) [file pone.0002484.s009.doc]

**Table S3**. Primer sequences used in real time PCR

Gene ID Forward (5'-3') Reverse (5'-3')

MAL7P1.27 AGATTTCGTAACTTTGTTTGG ATCGACGTTGGTTAATTCTC

PF11_0265 AGCTCACCTTGGTTTATATTTAC AGCTATGGTATAGACAACTTC

PF14_0102 ATGGGTGGTGATGATCTTAT TAATACAGCAGCAATATCTTGAG

PF14_0399 GAGGAAAGATTATATGGATCTAC CATATTCATTTCGTTCAATTTGAG

PF14_0738 ACCTTGCCTATGGTTTAATG AGTAATGCATCTTCATATGTGC

PF14_0753 AGATCCTTAGCAGAATATGATAC AAAGATAGACCTTCATTGGTTTC

PFE0080c ATCTGAGAGAGAACATTTGG GGTGTATGAACTCTTGATCC

PFE0785c TCATCAAATCAAACAAGTCACG CTTGTGAATCAAGCATTTCATG

PFE1125w CAACTAGTTTACTGAGGTATG GTACTGCCTATTGTTATCATG

PFE1150w ACTATTGCCCACAGAATTGC CCATCTTGTGCTGATAATAATTC

MAL13P1.284 GTTGTGGACCTGCATATGTT TTGACCATTTCAACTGAACC

PFI0115c GGAGATATGCGTTTGTGTG TCCCAACACTAGGTATACACG

PFI0270w TAAATTTCAATGGTTAGATCC CTTCATCACTTTCTTTATCTTC

MAL13P1.287 ATGTTATCAAATGAAATGACTT ACGTAGTTGACGAACACATA

MAL13P1.480 GATAACAATAACTCCGAATTTAAC TGCTTGACTTTCGTGTAATAATC

MAL8P1.48 AGAGGCTATGACACATTTATGA CTTTGTCTAAACATTCCCAAT

PFL1700c ACAATGGGTATGCTTTCAAC TACATTACCAACGGCATCTA

PFI1685w TGATTGGTGGACTTTAGGTA CTTCATCAAATGTTTGCAATTG

PF11_0066 AAATATTAGCGACACCGAAC TGAATCTGAATCAACATCACTT

PF11_0370 AGTAATGCTACCATGCCAAT AAGGCCGTAAACCCAATATG

PFL1080c TATTTGTGGAGAAGGAGGAG TCTAATTTCCATTTCAAAGGC

PFF1430c TCAAAGCATGTTAGCTCTTG TCTGCATGTGTTTCTTGGTA

PF10_0016 GAAGATTTATTTCAAGCTTCTG ACAATTACCTACAGTACTTTGC

PF10_0019 TTCTCGCTGCCTTAATAGG GTGCTTCCTTCTTGTGATTTG

PF10_0020 AGCTGATGACAAGGATGTATC TCCATAAATGTTTGTCAGGTTC

PFB0340c GCAGGATCAGGACAAAGTG TTTACGCATACCCATTCTTA

MAL13P1.209 AATATCACATGGCCAATCACC CAATCTCTTATCATCTGTTATT

PFE1150w TGCATCTATAAAACGATCAGACAAA TCGTGTGTTCCATGTGACTGT

PF10-0084 TGATGTGCGCAAGTGATCC TCCTTTGTGGACATTCTTCCTC

MAL7P1.176 GTGTTGTGCAGTATCAGATT GAAAAAGCCTCCTTTCTGAA
